# Curriculum Vitae of WEE2 Kinase in Homeostasis and Diseases: A Systematic Review

**DOI:** 10.3390/cells15131147

**Published:** 2026-06-24

**Authors:** Ran Wang, Jing Yu, Yan-Jun Liu, Guo-Shu Zhao, Xiang Li, Yi-Fang Jiang, Chang-Hong Li, Guan-Jun Yang, Jiong Chen

**Affiliations:** 1 State Key Laboratory for Quality and Safety of Agro-Products, School of Marine Sciences, Ningbo University, Ningbo 315211, China; 2411130037@nbu.edu.cn (R.W.); 2411130055@nbu.edu.cn (J.Y.); 18851156219@163.com (Y.-J.L.); 18667910763@163.com (G.-S.Z.); 2511130038@nbu.edu.cn (X.L.); 2511130027@nbu.edu.cn (Y.-F.J.); lichanghong@nbu.edu.cn (C.-H.L.); 2 Laboratory of Biochemistry and Molecular Biology, School of Marine Sciences, Meishan Campus, Ningbo University, Ningbo 315832, China; 3 Key Laboratory of Aquacultural Biotechnology of Ministry of Education, Ningbo University, Ningbo 315211, China

**Keywords:** WEE2, cell cycle regulation, WEE2 mutations, reproductive diseases, inhibitors

## Abstract

**Highlights:**

**What are the main findings?**

- WEE2 is an oocyte-specific kinase essential for meiotic arrest and progression.
- WEE2 mutations are a major cause of total fertilization failure and female infertility.

**What are the implications of the main findings?**

- WEE2 structure, function, and mutational mechanisms lay a core molecular foundation for understanding associated infertility.
- Latest progress on WEE2 inhibitors offers new avenues for precise diagnosis and targeted treatment of reproductive disorders.

**Abstract:**

WEE2, an oocyte-specific kinase of the WEE family, is a core regulator of oocyte meiosis. It maintains germinal vesicle (GV) arrest and prevents premature meiotic resumption by phosphorylating cyclin-dependent kinase 1 (CDK1), thereby inhibiting maturation-promoting factor (MPF) activity. WEE2 also regulates exit from metaphase II (MII), ensuring orderly meiotic progression. Consequently, the functional integrity of WEE2 is essential for female reproduction. Homozygous or compound heterozygous mutations in the *WEE2* gene represent a major genetic cause of total fertilization failure and primary infertility, as these mutations lead to reduced or abolished kinase activity, impair meiotic control, and disrupt oocyte maturation and embryonic development. This review systematically summarizes the protein structure, core functions, and mutation types of WEE2, along with its association with total fertilization failure and female primary infertility. It also highlights research advances in WEE2-targeted inhibitors and discusses the potential applications and future directions of WEE2 in the diagnosis and management of reproductive disorders.

## 1. Introduction

WEE2 is an oocyte-specific tyrosine kinase belonging to the WEE kinase family, which also includes WEE1 (Wee1-like protein kinase) and PKMYT1 (membrane-associated tyrosine- and threonine-specific cyclin-dependent kinase 1 (CDK1)-inhibitory kinase, also known as MYT1) [1]. As dual-specificity protein kinases in the serine/threonine kinase superfamily, WEE family members function as core negative regulators of the G2/M cell cycle checkpoint by phosphorylating CDK1 and inhibiting maturation-promoting factor (MPF) activity, thereby temporally controlling eukaryotic cell division [2]. WEE1 and PKMYT1 regulate the somatic cell cycle; their inhibition causes premature CDK1 activation, leading to cell cycle dysregulation and genomic instability [2].

Unlike its homologs, WEE2 primarily governs oocyte meiosis: it maintains germinal vesicle (GV) arrest and regulates exit from metaphase II (MII) [3,4]. During the GV stage, WEE2 phosphorylates CDK1 at Tyr15 within the CDK1/cyclin B (MPF) complex, reducing MPF activity and sustaining meiotic arrest. Following an LH surge, cAMP degradation suppresses WEE2 activity, activates CDK1, and triggers GV breakdown (GVBD) and meiotic resumption [5,6]. In MII, WEE2 again inhibits MPF activity via the same mechanism, promoting oocyte exit and pronuclear formation. Pathogenic variants of *WEE2* are predominantly autosomal recessive meaning that the mutations must come from both parents to be phenotypically observable. Existing research indicates that pathogenic *WEE2* mutations occur at a frequency below 0.01% among the general population [7,8]. Homozygous or compound heterozygous mutations cause loss or marked reduction in WEE2 function, resulting in persistent MPF activation, premature GVBD, and failure of MII exit and pronuclear formation. This constitutes a major pathogenic mechanism underlying oocyte maturation defects (OMD), total fertilization failure (TFF), and primary infertility. Recent studies have identified *WEE2* as a causative gene for early embryonic arrest (EDA); mutations in its kinase domain block embryo development at the 2–4-cell stage, expanding the phenotypic spectrum of WEE2-related disorders and confirming WEE2 as a maternal effect gene with marked phenotypic heterogeneity [9].

Functional differences between WEE2 and WEE1/PKMYT1 arise from distinct expression patterns, substrate specificities, and functional localizations [10]. WEE2 is highly expressed during oocyte maturation but barely detectable in somatic cells, contrasting with the ubiquitous expression of WEE1 and PKMYT1 [11]. Non-human primate tissue profiling confirms predominant oocyte expression of WEE2, with low testicular and no detectable somatic expression [12]. However, a recent high-sensitivity proteomic study (LC–MS/MS) identified low-abundance WEE2 in mouse skeletal muscle, suggesting a conserved role in aerobic capacity and potential non-reproductive functions [13]. Regarding substrate specificity, WEE1 and WEE2 primarily target CDK1 Tyr15, whereas PKMYT1 phosphorylates both Thr14 and Tyr15 [12,14]. Functionally, WEE2 plays unique roles in germ cells under specific regulation by PKA, while WEE1 and PKMYT1 are more broadly involved in somatic cell cycle checkpoints and responses to DNA damage or developmental signals.

Mutations in *WEE2* disrupt oocyte meiotic regulation and pronuclear formation after fertilization, leading to irreversible fertilization failure and female infertility [15]. In vitro fertilization (IVF) is a current treatment approach. Studies show that microinjection of *WEE2* cRNA into human oocytes can effectively rescue fertilization function, offering a promising therapeutic strategy [16]. Recently, WEE2 has become a popular target in reproductive medicine and non-hormonal contraception research [14,15]. Selective inhibition of its kinase activity, based on WEE2 structural specificity, may serve as an effective non-hormonal contraceptive method. WEE1 and PKMYT1 inhibitors have been extensively developed as candidate chemotherapeutic agents for oncology [11,17]. As a representative WEE1 inhibitor, AZD1775 also non-specifically inhibits WEE2 due to conserved kinase domains, which results in severe off-target effects [18]. Therefore, novel WEE2 inhibitors with improved target specificity are urgently needed in clinical settings to treat a wide range of diseases caused by WEE2 dysfunction. Systematic reviews on WEE2 structure and function remain limited. This article systematically summarizes progress on the protein structure, biological functions, pathogenic mutations, associated reproductive diseases, and targeted inhibitors of WEE2. It further dissects the regulatory mechanisms of WEE2, aiming to provide genetic markers for diagnosing reproductive disorders and to lay a foundation for targeted therapy and novel contraceptive strategies.

## 2. Materials and Methods

### 2.1. Study Design and Reporting Guidelines

The design and drafting of this systematic review strictly adhere to the 2020 Preferred Reporting Items for Systematic Reviews and Meta-Analyses (PRISMA) statement. The primary objective of this review is to comprehensively collate evidence concerning WEE2, including its structural features, biological functions, mutation profiles and disease correlations, as well as preclinical and clinical research data of WEE2-targeted small-molecule inhibitors. This work further elaborates on the regulatory mechanisms of WEE2, aiming to identify genetic biomarkers for the diagnosis of reproductive system diseases and lay a theoretical foundation for targeted therapy and innovative contraceptive strategies.

### 2.2. Literature Search Strategy

This study performed comprehensive and independent electronic literature searches in PubMed to screen relevant investigations on the biological structure and function of the WEE2 protein, its gene mutations, and corresponding targeted inhibitors. The search scope included original research articles published between January 2000 and June 2026, and the complete retrieval procedure was finalized on 18 June 2026. In an initial search, a total of 82 search papers were identified in PubMed using the keyword search terms WEE2.

### 2.3. Eligibility Criteria

This study included all relevant publications released globally from 2000 to the present. The research scope covered comprehensive studies on the structural and functional features of WEE2 protein, pathogenic gene mutations, pathogenic mechanisms of female infertility, functional validation using gene-edited animal models, *WEE2* cRNA rescue assays, and the development of targeted small-molecule inhibitors. No restrictions were set for article types, formats or publication platforms, and all studies focusing on functional WEE2 protein were eligible for inclusion in this systematic analysis. By contrast, studies that only explored homologous kinases WEE1 and PKMYT1, non-coding RNAs such as *WEE2-AS1*, or other molecules without direct regulatory connections to WEE2 protein were excluded.

### 2.4. Study Selection

**Figure 1:**
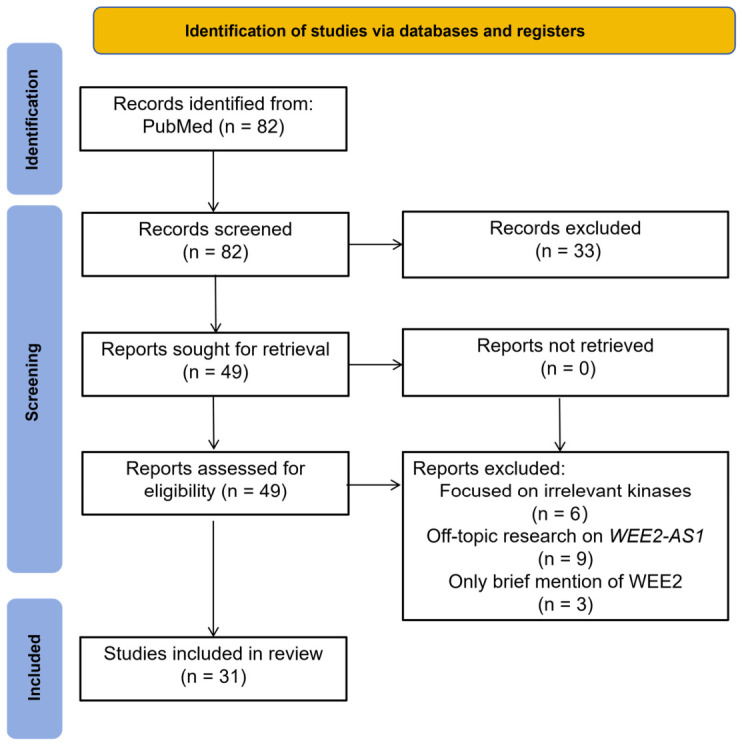
PRISMA flowchart. Flow diagram summarizing the performed systematic review.

All retrieved records were deduplicated by automatic software matching together with manual verification. Two researchers independently conducted two rounds of screening: they first performed preliminary screening by titles and abstracts and then retrieved full texts of potentially relevant articles to assess eligibility. Any disagreements between the two reviewers were discussed with a third senior researcher until a consensus was reached. A flow diagram complying with the PRISMA 2020 guidelines (Figure 1) was plotted to count the number of retained and excluded records at each screening stage and specify all reasons for exclusion.

### 2.5. Data Extraction

Extracted data fell into three categories: clinical data of *WEE2* variants in infertile cohorts, cellular and animal data with *WEE2* knockout or overexpression, and experimental results of WEE2 kinase inhibitors. Key extracted information included study profiles, variant characteristics, model construction protocols, reproductive phenotypes and functional parameters of inhibitors. Discrepancies were resolved by rechecking full articles; no subjective speculation was made for incomplete data reported in included studies.

### 2.6. Data Synthesis

Included studies exhibited substantial heterogeneity in participant cohorts, *WEE2* gene manipulation protocols, oocyte experimental procedures and chemical structures of inhibitors; therefore, quantitative meta-analysis was not performed. We synthesized evidence via qualitative narrative synthesis covering three themes: clinical data of *WEE2* variants linked to infertility, relevant disease phenotypes, and preclinical studies on selective WEE2 inhibitors. All experimental and clinical findings were summarized qualitatively without a unified table of included studies.

### 2.7. Risk of Bias/Methodological Quality Assessment

Included studies encompassed human clinical sequencing cohorts, gene-edited animal experiments, and in vitro and in vivo functional assays of WEE2 kinase inhibitors. There was substantial heterogeneity across diverse study designs, and no universal standardized quality assessment tool was available. Accordingly, standardized risk-of-bias assessment was not performed in this review.

## 3. Structure of WEE2

### 3.1. Gene Location and Evolutionary Conservation of WEE2

**Figure 2:**
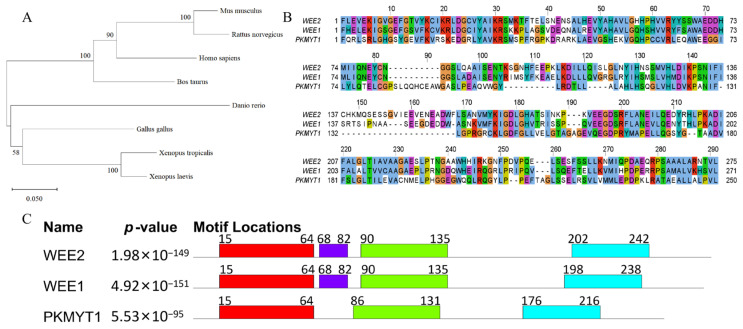
Evolutionary conservation and sequence analysis of WEE2 kinase domain. (**A**) Phylogenetic tree of WEE2 kinase domain sequences from different species. Analysis and tree construction were performed using MEGA12 software. Node values represent bootstrap support (%), indicating the reliability of evolutionary branches. (**B**) Multiple sequence alignment results of the kinase domains of WEE2, WEE1 and PKMYT1. This figure was constructed using the MAFFT tool in Jalview software with the classic Clustal color scheme. Different colors correspond to amino acids with distinct physicochemical properties, and darker shading indicates higher sequence conservation at this site. (**C**) Conserved motif analysis of the kinase domains of WEE2, WEE1 and PKMYT1. Analysis in this figure was performed using the MEME online suite. Different colors represent distinct conserved motifs.

Each member of the WEE family is distributed on distinct chromosomes. The *WEE2* gene (also known as *WEE1B*) is located on human chromosome 7. According to the NCBI database, its full-length genomic sequence spans 22,919 nucleotides (nt) and comprises 12 exons. The canonical mRNA transcript is 3061 nt in length, encoding a protein of 567 amino acids (aa). As an oocyte-specific cell cycle regulatory kinase, WEE2 exhibits a highly evolutionarily conserved kinase domain across species from lower vertebrates (*Xenopus laevis*) to higher mammals (*Homo sapiens*), with >93% sequence identity among closely related species, indicating essential and irreplaceable biological functions (Figure 2A). Multiple sequence alignment using Jalview (v2.11.5.1) revealed that the kinase domain of human WEE2 shares 63.27% identity with WEE1, markedly higher than the 33.7% identity with PKMYT1, suggesting a closer phylogenetic relationship between WEE2 and WEE1. Nevertheless, the core functional motifs within the kinase domains remain highly conserved across all three proteins, reflecting the functional constraint characteristic of the protein kinase family (Figure 2B). Homologous motif prediction using the MEME Suite (v5.5.9) identified four highly significant conserved motifs (*p* < 1 × 10^−90^) in human WEE2 and its family kinases. WEE2 shares all four motifs with WEE1, whereas PKMYT1 shares only motifs 1, 2, and 3. These results support the homologous evolutionary relationship among the three kinases and underscore the functional conservation of CDK1 phosphorylation and cell cycle arrest mediated by their conserved domains (Figure 2C).

### 3.2. Domain Architecture and Protein Sequence Features of WEE2

WEE2 is a 567-amino-acid (aa) protein, whereas WEE1 and PKMYT1 comprise 646 aa and 499 aa, respectively. Jalview alignment [19] shows that the overall sequence identity among the three proteins is considerably lower than within the kinase domain: 44.98% between WEE2 and WEE1, 26.39% between WEE2 and PKMYT1, and 26.65% between WEE1 and PKMYT1. Thus, functional divergence among the three kinases arises not only from subtle differences within the kinase domain but also largely from distinct sequences and phosphorylation sites in the N- and C-terminal regions.

**Figure 3:**
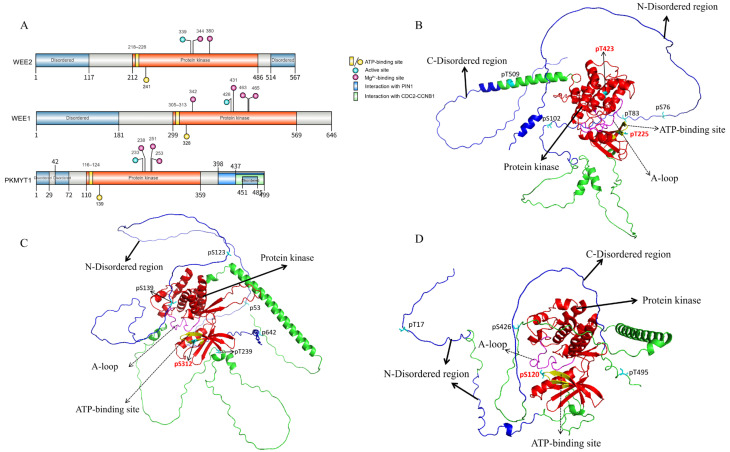
Domain architecture and 3D structural mapping of WEE2, WEE1 and PKMYT1 kinases. (**A**) Comparative analysis of protein structural characteristics among the three members of the WEE kinase family. Data in this figure were retrieved from the UniProt database and visualized using the IBS 2.0 online server. (**B**) Schematic diagram of the tertiary structure of WEE2. (**C**) Schematic diagram of the tertiary structure of WEE1. (**D**) Schematic diagram of the tertiary structure of PKMYT1. (**B**–**D**) were predicted by AlphaFold2 and visualized using PyMOL (3.1.6.1). Functional regions including N-terminal/C-terminal disordered regions, kinase domain, activation loop, ATP-binding sites and selected phosphorylation sites are highlighted in different colors with corresponding annotations.

Experimental 3D structures of catalytic domains from human WEE kinases are all derived from conventional X-ray crystallography, and no resolved structures exist for their N- and C-terminal regulatory regions [18]. Domain annotations from UniProt (Figure 3A) reveal marked divergence in N-/C-terminal sequence features distribution among WEE family members, forming the structural basis for their functional specificity. WEE2 contains intrinsically disordered regions (IDRs) at both the N-terminus (residues 1–117) and C-terminus (514–567), linked to the central kinase domain by flexible linkers. In contrast, WEE1 possesses a longer N-terminal IDR but no C-terminal IDR. PKMYT1 harbors two short N-terminal IDRs, one C-terminal IDR, and additional interaction regions for PIN1(Peptidylprolyl Cis/Trans Isomerase, NIMA-Interacting 1) [20] and the CDK1–cyclin B complex. PIN1 interacts with the C-terminal domain of Myt1 in a phosphorylation-dependent way. These differences collectively constitute critical molecular evidence for the functional divergence of WEE kinases.

A search of the PhosphoSitePlus database [21] (https://www.phosphosite.org/, accessed on 23 May 2026) revealed a total of 6 phosphorylation sites on the full-length human WEE2 protein that have been verified by both high-throughput mass spectrometry and low-throughput experiments. Among these, T225 and T423 are located within the kinase domain (Figure 3B). Alignment with the UniProt database confirmed that T225 is homologous to S312 of WEE1 and S120 of PKMYT1, both of which reside within the ATP-binding pocket. Phosphorylation at this site may regulate kinase catalytic activity by modulating ATP binding efficiency. Prediction results from the NetPhos 3.1 server [22] (https://services.healthtech.dtu.dk/services/NetPhos-3.1/, accessed on 23 May 2026) indicated that high-confidence phosphorylation sites (>0.7 threshold) in WEE2 are highly enriched in the N- and C-terminal IDRs, and are specifically recognized only by PKA and PKC, demonstrating high kinase specificity. In contrast, WEE1 is recognized by a broader spectrum of kinases, including PKA, PKC, CDK5, CKII, and PKB. PKMYT1 is primarily targeted by PKA, PKC, and PKB, exhibiting intermediate kinase specificity between WEE2 and WEE1.

### 3.3. WEE2 Secondary and Tertiary Structures

AlphaFold2 prediction combined with ESPript 3.2 visualization shows that the core α-helices and β-sheets in the kinase domains of WEE2, WEE1, and PKMYT1 are highly conserved in arrangement. The ATP-binding region in all three proteins adopts a β-sheet scaffold, reflecting conserved catalytic function within the family. Full-length WEE2 contains 12 α-helices, 4 3_10_-helices (η), 9 β-sheets, and several random coils. The core kinase domain forms a stable catalytic scaffold, whereas the N- and C-terminal random coil regions serve as independent regulatory modules, providing sufficient binding space for PKA/PKC-mediated reproductive-specific phosphorylation. This is highly consistent with the terminal regulatory features predicted by UniProt and NetPhos 3.1, together constituting the structural basis for WEE2-specific regulation of oocyte meiosis.

The full-length tertiary structure of WEE2 features a compact core and flexible termini. Its kinase domain adopts a classic bilobal folding pattern: a small N-terminal subdomain and a large C-terminal subdomain linked by a flexible hinge, forming a catalytic pocket for ATP binding and substrate phosphorylation. Random coils appear as flexible disordered arms attached to the kinase domain, capable of regulating its activity through spatial conformational changes. The small N-terminal subdomain contains the ATP-binding motif and key salt bridges that stabilize the ATP-bound conformation. The large C-terminal subdomain houses the substrate-binding region, active site, magnesium ion-binding site, and activation loop; conserved residues in the catalytic loop promote phosphate transfer. Compared with WEE1, WEE2 possesses a shorter and conformationally more stable N-terminal random coil region, which prevents interference from non-specific signals. Unlike the membrane-localized PKMYT1, full-length WEE2 contains no transmembrane domain; its N- and C-terminal flexible disordered arms can swing freely, giving WEE2 a typical soluble protein conformation (Figure 3B–D). All above structural comparisons and phosphorylation predictions are derived from public bioinformatic webservers and the precomputed AlphaFold2 database, rather than original experimental structural data generated in this study.

In summary, structural differences between WEE2 and WEE1/PKMYT1 are mainly concentrated in reproduction-specific regulatory terminal amino acid sequences, secondary structure diversity, tissue specificity of substrate recognition, and inhibitor-binding structures. These differences likely arise from key residue mutations or local conformational changes, and they underpin the exclusive role of WEE2 in oocyte meiosis regulation. This makes WEE2 a specific target for the diagnosis and treatment of reproductive diseases, highlighting its unique biological value [23].

## 4. WEE2 Regulatory Network and Signaling Pathways

**Figure 4:**
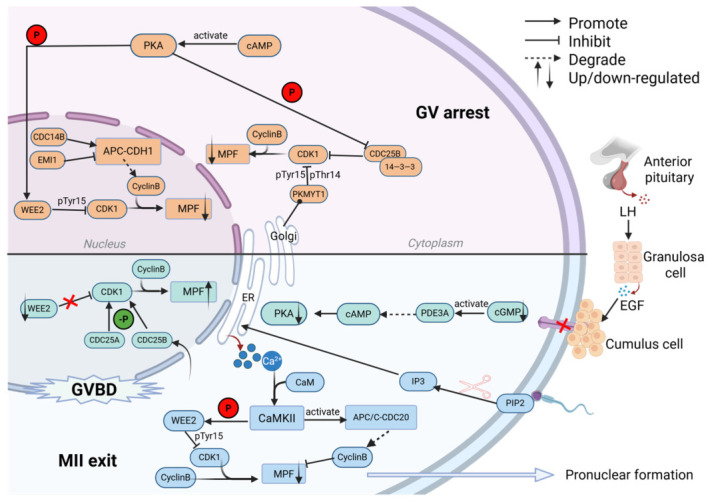
Regulatory network and signaling pathways of WEE2. This figure depicts the core regulatory network of WEE2, including its upstream regulators, downstream target molecules, and the major signaling pathways involved in WEE2-mediated biological processes. Abbreviations: Protein Kinase A (PKA), Cyclic Adenosine Monophosphate (cAMP), Cyclic Guanosine Monophosphate (cGMP), Phosphodiesterase 3A (PDE3A), Calmodulin (CaM), Calcium/Calmodulin-Dependent Protein Kinase II (CaMKII), Inositol 1,4,5-trisphosphate (IP3), Phosphatidylinositol 4,5-bisphosphate (PIP2), Epidermal Growth Factor (EGF), Luteinizing Hormone (LH), Cell Division Cycle 25 Homolog A (CDC25A), Cell Division Cycle 25 Homolog B (CDC25B), Cell Division Cycle 14 Homolog B (CDC14B), Anaphase-Promoting Complex–Cdc20 Homolog 1 (APC–CDH1), Anaphase-Promoting Complex/Cyclosome–CDC20 (APC/C–CDC20), Early Mitotic Inhibitor 1 (EMI1), Tyrosine 3-Monooxygenase/Tryptophan 5-Monooxygenase Activation Protein (14-3-3), Endoplasmic Reticulum (ER), Golgi Apparatus (Golgi). P represents phosphorylation, and -P represents dephosphorylation.

WEE2 does not function independently but is embedded in multiple highly conserved signaling pathways to form a precise regulatory network that collectively governs the arrest, maintenance and resumption of meiosis in oocytes (Figure 4). In mouse oocytes, WEE2 and PKMYT1 jointly maintain GV stage arrest: WEE2 inhibits nuclear CDK1, whereas PKMYT1 inhibits cytoplasmic CDK1, establishing a dual inhibitory mechanism. WEE2 also antagonizes CDC25 phosphatases: nuclear localized CDC25A and cytoplasm nucleus shuttling CDC25B can relieve the inhibition by WEE2 through dephosphorylating CDK1, thus driving the resumption of meiosis. In addition, the APC–CDH1 complex prevents premature CDK1 activation by degrading cyclin B1 and blocking its binding to CDK1. CDC14B phosphatase maintains APC–CDH1 activity (Anaphase-Promoting Complex–Cdc20 Homolog 1, a core E3 ubiquitin ligase, maintains GV arrest in oocytes), while EMI1 (Early Mitotic Inhibitor 1, an oocyte-specific endogenous inhibitor of APC–CDH1) inhibits its activity to balance cell cycle progression [5]. In the regulation of prophase of meiosis, the cAMP/PKA signaling pathway acts as the core upstream pathway [12], a mechanism clearly demonstrated in rhesus macaques: high intracellular levels of cAMP activate PKA, which in turn stabilizes and activates WEE2 via phosphorylation [1,24]. Activated WEE2 continuously phosphorylates the Tyr15 site of CDK1, suppresses the activity of MPF, maintains oocyte arrest at the GV stage, and prevents precocious meiotic entry. Meanwhile, PKA can phosphorylate CDC25B, causing it to bind 14-3-3 proteins and be retained in the cytoplasm [25].

Studies have shown that the surge of luteinizing hormone (LH) in vivo serves as a key signal for the resumption of GV stage arrest in mouse oocytes [6]. LH initiates a paracrine signaling pathway in oocytes by inducing granulosa cells to secrete EGF like factors, which closes gap junctions between oocytes and cumulus cells and lowers intracellular cGMP levels in oocytes. This in turn activates PDE3A to degrade cAMP and reduces PKA activity, thereby indirectly releasing meiotic arrest. In vitro experiments have confirmed that when oocytes are removed from follicles, intracellular cAMP spontaneously declines, directly leading to attenuated PKA activity. This results in decreased phosphorylation of WEE2 and nuclear translocation of CDC25B; once CDK1 is activated, meiosis resumes [5].

The CaMKII pathway mediates the exit of oocytes from MII arrest [16,26]. Sperm–oocyte fusion triggers intracellular Ca^2+^ oscillations, activating CaMKII. Activated CaMKII directly phosphorylates Ser15 of WEE2 and enhances its kinase activity [27], activated WEE2 phosphorylates and inhibits CDK1, lowering MPF activity. The downstream APC/C-CDC20 pathway is then activated, degrading cyclin B and inactivating MPF. MPF inactivation completes meiosis, extrudes the second polar body, and forms pronuclei, accomplishing fertilization [26]. When WEE2 function is lost or reduced, CDK1 remains uninhibited, causing persistent MPF activation. Even if sperm entry triggers Ca^2+^ oscillations, defective WEE2-mediated signal conversion prevents MPF inactivation, ultimately leading to MII exit failure and fertilization arrest.

These pathways and interacting molecules collectively form a WEE2-centered meiotic regulatory module that ensures the proper timing, fidelity, and developmental competence of oocyte maturation.

## 5. Biological Functions of WEE2

**Figure 5:**
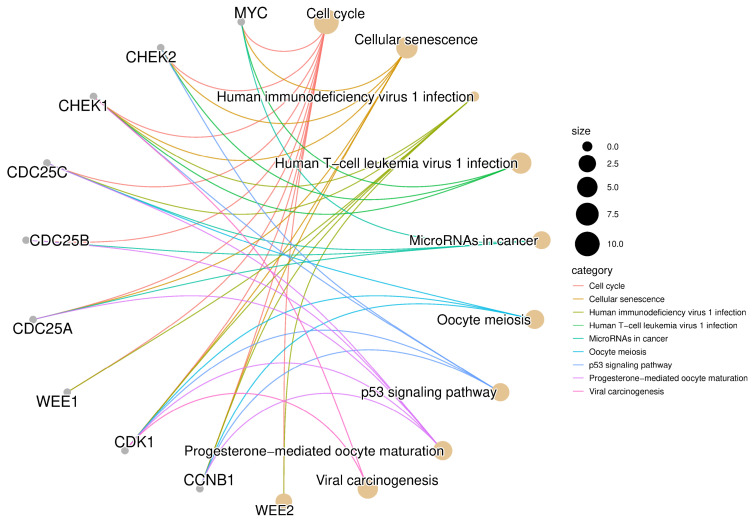
Network analysis of WEE2-interacting proteins and their associated KEGG pathways. This figure demonstrates that these genes are predominantly enriched in the oocyte meiosis, progesterone-mediated oocyte maturation, cell cycle, and p53 signaling pathways, constituting a core molecular network regulating oocyte meiotic arrest and resumption. Interacting protein data were derived from STRING (https://string-db.org, accessed on 23 May 2026) and BioGRID (https://thebiogrid.org, accessed on 23 May 2026). Heat maps were plotted using the Bioinformatics Tools Platform (https://www.bioinformatics.com.cn, accessed on 23 May 2026). Abbreviations: MYC proto-oncogene (MYC), Checkpoint kinase 1 (CHEK1), Checkpoint kinase 2 (CHEK2), Cell division cycle 25 homolog C (CDC25C), Cyclin B1 (CCNB1).

**Figure 6:**
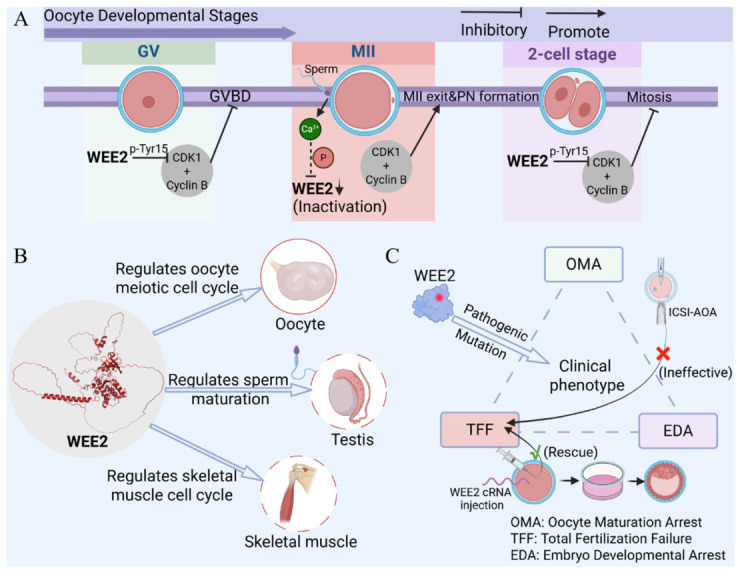
WEE2 regulatory network, physiological functions, and clinical consequences of pathogenic mutations. (**A**) Regulatory function of WEE2 during oocyte development. (**B**) Systemic physiological functions of WEE2 in multiple tissues. (**C**) Clinical phenotypes and targeted interventions of pathogenic WEE2 mutations. Abbreviations: Germinal vesicle (GV), Metaphase II (MII), Cyclin-dependent kinase 1 (CDK1), Pronuclear stage (PN).

As an oocyte-specific protein kinase, WEE2 functions as a molecular switch for meiotic progression. It exhibits strict tissue specificity: high expression in the ovary (oocytes), low expression in the testis, and negligible expression in somatic tissues. KEGG pathway enrichment analysis indicates that WEE2 and its interacting proteins cooperatively regulate core reproductive pathways, such as oocyte meiosis and progesterone-mediated oocyte maturation, thereby ensuring precise control of the cell cycle and reproductive development (Figure 5). WEE2 localizes to the nucleus at the GV stage and disperses into the cytoplasm following GVBD [12]. It is continuously expressed throughout the GV, MI, and MII stages (Figure 6), declines after fertilization, and becomes undetectable in early embryonic development. Thus, WEE2 functions across the entire process from oocyte maturation to fertilization.

### 5.1. Dual Regulation of Oocyte Meiosis

During the GV stage, WEE2 phosphorylates CDK1 at Tyr15, thereby inhibiting CDK1 (a core subunit of MPF) and suppressing MPF activity. This arrests the cell cycle at the GV stage and prevents premature GVBD [3,4,5]. This process is coordinately regulated by the cAMP–PKA axis and the APC–CDH1 complex. Following a gonadotropin surge or a decrease in intracellular cAMP, the inhibitory effect of WEE2 is relieved, promoting meiotic resumption. At the MII stage, WEE2 serves as the core negative regulator maintaining meiotic arrest. Sperm-triggered Ca^2+^ oscillations inactivate and degrade WEE2, relieving its inhibition of CDK1 and leading to MPF inactivation. This ultimately promotes oocyte exit from MII, second polar body extrusion, and pronuclear formation, completing fertilization [26]. Functional abnormalities in WEE2 prevent oocyte exit from MII, resulting in fertilization arrest.

### 5.2. Factors Affecting Early Embryonic Development

WEE2 is a key regulatory molecule linking oocyte maturation and early embryonic development, playing an irreplaceable role in fertilization and the initiation of embryogenesis. In rhesus monkeys, WEE2 is significantly degraded after fertilization and becomes undetectable in the embryo [12]. Extensive WEE2 degradation relieves CDK1 inhibition, initiating the first zygotic mitosis and marking the official start of embryonic development. Insufficient WEE2 degradation leads to persistent CDK1 inhibition, preventing normal mitotic initiation and causing embryonic arrest. Multiple studies have shown that homozygous or compound heterozygous mutations in *WEE2* result in marked absence or reduction in WEE2 expression. While most cases present with complete fertilization failure (no 2PN formation), a small subset of patients exhibit a very low rate of 2PN formation; however, all embryos show fragmented developmental arrest and fail to implant normally [28,29]. Neither conventional intracytoplasmic sperm injection (ICSI) nor artificial oocyte activation (AOA) improves fertilization outcomes [7].

### 5.3. Functions of WEE2 in Testis

According to the Genotype–Tissue Expression (GTEx) project, *WEE2* is expressed in the testis and may downregulate spermatocyte meiotic progression during spermatogenesis. Studies have shown that a bitter taste receptor gene polymorphism in Caucasian men is associated with male infertility. The minor allele of *TAS2R3-rs11763979* is associated with a decrease in the number of sperm with a normal morphology and with an increase in *WEE2-AS1* expression. It is plausible to hypothesize that the increased *WEE2-AS1* expression may downregulate *WEE2*, which in turn can alter the natural timing of sperm maturation, increasing the number of abnormal sperm cells [30].

### 5.4. Potential Functions of WEE2 in Skeletal Muscle

Beyond its reproductive roles, it is speculated that WEE2 may also possess non-reproductive functions. WEE2 was significantly enriched in a study investigating the association between aerobic capacity (AC) and the skeletal muscle proteome, where it was negatively correlated with AC and identified as a cross-model conserved associated protein. Pathway enrichment analysis revealed that in wild-type mice, WEE2 is enriched in the “cell cycle regulation” pathway, whereas in McArdle disease mice, it is enriched in “organic nitrogen compound metabolism” and “protein metabolism” pathways and classified under “phosphoprotein.” It is speculated that WEE2 may regulate phosphorylation of proteins involved in skeletal muscle cell cycle or metabolism [13]. This function awaits validation by in vivo and in vitro experiments but provides a new direction for expanding the tissue expression profile and functional scope of WEE2.

## 6. WEE2 and Diseases

### 6.1. Oocyte Maturation Defect (OMD)

OMD is a major cause of primary infertility in women, characterized by failure of oocytes to complete meiotic maturation either in vivo or during in vitro culture, thereby preventing normal fertilization. Monogenic factors are important pathogenic contributors, and *WEE2* is one of the core causative genes [31]. Pathogenic mutations in *WEE2* produce non-functional or functionally impaired truncated proteins, leading to defective kinase activity [32]. This results in premature GVBD or persistent MII arrest, ultimately causing asynchrony between nuclear and cytoplasmic maturation.

### 6.2. Total Fertilization Failure (TFF)

TFF is a serious complication in IVF and ICSI cycles, referring to failure of all mature oocytes to fertilize normally, with an incidence of 1–3% [33]. Its core etiology is oocyte activation defect (OAD), which can be sperm- or oocyte-related; *WEE2* is the core oocyte-related pathogenic gene [34]. Clinical studies report a *WEE2* mutation frequency of 6–20.8% in TFF patients [16]. Compound heterozygous mutations (e.g., c.1535+3A>G/c.946C>T) lead to loss of WEE2 function and continuous MPF activation. Even ICSI combined with artificial oocyte activation (ICSI-AOA) using calcium ionophore cannot effectively activate the ooplasm or form pronuclei, resulting in fertilization failure [28,35]. A prospective controlled study showed that the rs1476640 polymorphism in *WEE2* is significantly associated with fertilization failure in women undergoing ICSI. The C allele increases risk (OR = 9.06, 95% CI: 3.27–25.14), while the T allele is protective. This polymorphism can be used to screen high-risk patients and guide personalized treatment strategies [36].

### 6.3. Early Embryonic Development Arrest (EDA)

EDA is a key cause of embryo transfer failure and reduced clinical pregnancy rates in IVF/ICSI cycles. It refers to developmental stagnation at a specific stage (typically 2- to 8-cell), preventing progression to blastocyst and implantation. Abnormalities in maternal effect genes, including WEE2, are core pathogenic factors. The detection rate of WEE2-related pathogenic mutations is approximately 5.95%, with embryos arresting at the 2–4-cell stage [9]. WEE2 abnormalities can lead to OMD, indirectly inducing fertilization disorders and EDA. Moreover, low-level residual expression of WEE2 in early post-fertilization embryos directly participates in regulating the MPF signaling pathway. Abnormal WEE2 expression or function disrupts MPF signaling, leading to developmental arrest.

All these diseases stem from pathological alterations in the *WEE2* gene (Figure 6) and are primarily associated with human reproductive health, especially female fertility. The far higher detection rate of *WEE2* pathogenic variants in infertile patients relative to healthy individuals highlights its essential function in governing the full reproductive cascade—oocyte maturation, fertilization activation, and early embryonic development—providing a basis for genetic diagnosis and treatment of infertility.

## 7. *WEE2* Mutations

**Figure 7:**
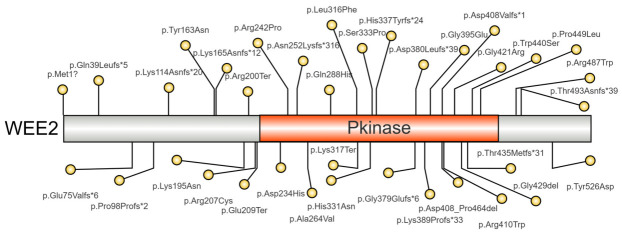
Lollipop diagram of pathogenic variant residue distribution in human WEE2 protein. The red shaded region represents the core protein kinase catalytic domain. Yellow circles mark the positions of amino acid alterations. The * represents a stop codon.

The protein encoded by *WEE2* is a key kinase regulating oocyte meiosis. At present, whole-exome sequencing (WES) is widely used to identify pathogenic mutations in infertile female patients with a variety of specific phenotypes, such as oocyte maturation defects [37] and empty follicle syndrome [38,39], while not a universal routine assay applicable to all infertile patients [40], WES serves as a critical step in etiological diagnosis. Accumulating evidence confirms that *WEE2* mutations play a critical role in oocyte maturation, fertilization, and early embryonic development, making it an important genetic cause of female infertility. Affected patients typically present with recurrent or complete fertilization failure, whereas heterozygous carriers generally show no clinical phenotype. Most *WEE2* mutations are pathogenic, usually occurring as homozygous or compound heterozygous variants following an autosomal recessive inheritance pattern. The main mutation types include frameshift, nonsense, missense, start codon, and splice site mutations (Table 1). All relative positions of variants are shown in the lollipop diagram (Figure 7). According to ACMG criteria, most are classified as “pathogenic” or “likely pathogenic”.

The kinase activity and physiological function of WEE2 depend critically on its intact spatial conformation, particularly the correct folding and arrangement of the kinase domain, ATP-binding pocket, nuclear export sequence (NES), catalytic loop, and substrate-binding region. Most pathogenic mutations disrupt protein conformation integrity, directly or indirectly, leading to loss of kinase function. The reversibility of such conformational alterations is markedly site-dependent and mutation-type-specific. Different mutation types cause distinct aberrant conformations, all ultimately abolishing or significantly reducing kinase activity, primarily through disruption of the ATP-binding site, substrate-binding region, or autophosphorylation capacity.

Missense mutations induce local conformational disturbances and spatial rearrangements. For example, c.619C>T (p.R207C) destabilizes local conformation and impairs kinase domain function [8]; c.1346C>T (p.P449L) reduces cavity volume within the kinase domain [28], interfering with ATP binding or CDK recognition; c.946C>T occurs in the protein kinase domain and NES [16], potentially disrupting both kinase conformation and nuclear-cytoplasmic shuttling. Frameshift and nonsense mutations cause protein truncation and complete loss of native conformation. The nonsense mutation c.949A>T [28] and the frameshift mutation c.1006_1007dup14 introduce premature stop codons, leading to early translational termination and deletion of core structures such as the kinase domain. Splicing mutations in *WEE2* cause domain deletions and abnormal conformations. The c.1222-1G>A variant leads to exon 9 loss in mRNA, producing an in-frame deletion of 57 amino acids in the kinase active domain, directly disrupting structural integrity and CDK1 phosphorylation function [32]. Additionally, several mutations induce aberrant subcellular localization. Missense mutations c.585G>C [7] and c.1319G>C [41] do not directly damage core kinase domain conformation but alter overall spatial folding, shifting WEE2 localization from nuclear to nucleocytoplasmic distribution, thereby abolishing its specific regulation of nuclear CDK1 and indirectly causing functional loss.

**Table 1:** Summary of the Types and Functional Consequences of *WEE2* Gene Mutations.

Location	Sequence Variation	Amino Acid Change	Mutation Type	Result	Reference
Exon 1	c.220_223delAAAG	p.Glu75Valfs*6	Frameshift deletion	WEE2 degradation	[26,35,42]
Exon 4	c.700G>C	p.Asp234His	Missense	WEE2 degradation	[35]
Exon 6	c.1006_1007insTA	p.His337Tyrfs*24	Frameshift insertion	WEE2 degradation	[35]
Exon 5	c.864G>C	p.Gln288His	Missense	Amino acid substitution	[14]
Exon 1	c.1A>G	p.Met1?	Initiation codon variant	Disruption of the start codon	[14]
Exon 4	c.619C>T	p.Arg207Cys	Missense	Disrupts the hydrogen bond and impairs the α-helix conformation	[8]
Exon 9	c.1228C>T	p.Arg410Trp	Missense	Alters hydrogen bonds between residues in the secondary structure	[15,26]
Exon 11	c.1576T>G	p.Tyr526Asp	Missense	May interfere with the C-terminal structure of WEE2 and reduce its function.	[14]
Exon 9	c.1261G>A	p.Gly421Arg	Missense	May impair the kinase domain and abolish its function.	[14]
Exon 4	c.725G>C	p.Arg242Pro	Missense	Impair the kinase domain and reduce its activity	[26]
Exon 6	c.997T>C	p.Ser333Pro	Missense	Impair the kinase domain and reduce its activity	[26]
Exon 6	c.991C>A	p.His331Asn	Missense	Impair the kinase domain and reduce its activity	[14]
Exon 8	c.1184G>A	p.Gly395Glu	Missense	Impair the kinase domain and reduce its activity	[26]
Exon 9	c.1319G>C	p.Trp440Ser	Missense	Abnormal subcellular localization and reduced WEE2	[41]
Exon 9	c.1286_1288delGAG	p.Gly429del	In-frame deletion	Decreased WEE2	[26]
Exon 10	c.1473dupA	p.Thr493Asnfs*39	Frameshift insertion	Decreased WEE2	[35]
Exon 6	c.949A>T	p.Lys317Ter	Nonsense	Decreased WEE2	[28]
Exon 9	c.1346C>T	p.Pro449Leu	Missense	Decreased WEE2	[28]
Exon 1	c.115_116insT	p.Gln39Leufs*5	Frameshift insertion	Decreased WEE2	[7]
Exon 4	c.756_758delTGA	p.Asn252Lysfs*316	Frameshift deletion	Decreased WEE2	[7]
Exon 10	c.1459C>T	p.Arg487Trp	Missense	Decreased WEE2	[7]
IVS 10	c.1535+3A>G	p.?	Splicing	Impairs RNA splicing	[16]
Exon 8	c.1221G>A	p.Asp408Valfs*1	Splicing variant	Disrupt RNA splicing; Produce truncated WEE2	[26,28]
Exon 9	c.1222-1G>A	p.Asp408_Pro464del	Splicing	Results in exon 9 deletion of WEE2 mRNA	[32]
IVS 4	c.759-2A>G	p.?	Splicing	Disrupts the normal splicing site	[43]
Exon 6	c.946C>T	p.Leu316Phe	Missense	May affect WEE2 phosphorylation and its nuclear localization	[16]
Exon 3	c.585G>C	p.Lys195Asn	Missense	Induces enhanced nuclear export of WEE2	[15]
Exon 2	c.487T>A	p.Tyr163Asn	Missense	Decreased WEE2 kinase activity	[9]
Exon 9	c.1304_1307delCCAA	p.Thr435Metfs*31	Frameshift deletion	CDK1 phosphorylation fails	[14]
Exon 5	c.791C>T	p.Ala264Val	Missense	Decreased pY15 of CDK1	[44]
Exon 8	c.1165_1168delAAAC	p.Lys389Profs*33	Frameshift deletion	May produce truncated WEE2 and disrupt protein structure	[9]
Exon 1	c.293_294insCTGAGACACCAGCCCAACC	p.Pro98Profs*2	Frameshift insertion	Produce truncated WEE2	[14]
Exon 4	c.598C>T	p.Arg200Ter	Nonsense	Produce truncated WEE2	[26,41]
Exon 1	c.341_342delAA	p.Lys114Asnfs*20	Frameshift deletion	Produce truncated WEE2	[14]
IVS 7	c.1136-2A>G	p.Gly379Glufs*6/p.Asp380Leufs*39	Splicing	Produce truncated WEE2	[15]
Exon 6	c.1006_1007dup	p.His337Tyrfs*24	Frameshift duplication	Produce truncated WEE2	[15]
Exon 4	c.625G>T	p.Glu209Ter	Nonsense	Produce truncated WEE2	[43]
Exon 3	c.495del ^a^	p.Lys165Asnfs*12	Frameshift deletion	Produce truncated protein	[45]

All mutations were identified in females with primary infertility, including novel private variants from individual patients and recurrent mutations detected across independent infertility cohorts. Variants were preliminarily screened via WES and further verified by Sanger sequencing. ^a^ Relevant nucleotide information was not reported in the original text. WEE2: WEE2 protein. The * represents a stop codon.

## 8. WEE2 Inhibitors for Non-Hormonal Female Contraception

Current oral emergency contraceptives are predominantly hormone-based (e.g., levonorgestrel and ulipristal acetate), which must be administered before the LH surge to block ovulation, have no effect on fertilization or implantation, and disrupt menstrual cycle timing [46]. Therefore, developing a non-hormonal, on-demand contraceptive that inhibits fertilization without disturbing the menstrual cycle represents a breakthrough for women’s autonomous fertility control.

Kinases are highly druggable; their activity can be modulated by small-molecule drugs. Humans encode 518 kinases across seven groups [47]. Most kinase-targeted drugs are Type I inhibitors that bind the ATP-binding pocket, with selectivity enhanced by unique amino acid sequences; allosteric modulators have also emerged. WEE2, an oocyte-specific kinase, uniquely suppresses meiosis during oocyte maturation initiation and regulates meiosis at fertilization. Existing research indicates that WEE2 rescues the lethal phenotype of WEE1 mutants in fission yeast, and WEE2 overexpression induces cell cycle arrest at the G2/M transition. Accordingly, we speculate that WEE2 overexpression may affect somatic cell mitosis, which offers theoretical support for developing WEE2 inhibitors (instead of structural analogs) to inhibit fertilization [48,49]. Selective WEE2 inhibitors exert contraceptive activity without affecting embryonic development or somatic cell function, a non-hormonal mechanism validated in mouse and non-human primate models [12,24].

Beyond conventional high-throughput screening (HTS), virtual HTS (vHTS) evaluates small-molecule binding via computational simulation, enabling rapid identification of active compounds. Combining vHTS with stepwise in vitro functional assays allows selection of selective WEE2 inhibitors [46,49]. Based on mechanism, WEE2 inhibitors fall into two categories: allosteric inhibitors (bind outside the ATP pocket, alter conformation, higher selectivity, less ATP competition) and ATP-competitive inhibitors (occupy the ATP pocket, block catalysis, most common type) (Table 2).

**Table 2:** Information Related to WEE2 Inhibitor.

Number	Name	Structure	WEE2 ΔTm (°C)	WEE1 ΔTm (°C)	PKMYT1 ΔTm (°C)	WEE2 IC50 (μM)	WEE1 IC50 (μM)	WEE2 Inhibition Rate (%)	WEE1 Inhibition Rate (%)	Embryonic Cleavage Rate (%)	HEK293 Cells Proliferation Inhibition	Reference
1	GPHR-00336382	Not disclosed	3.5	-	-	5.8 ± 1.2	>100	≥80	~0	-	-	[4]
2	GPHR-00355672	Not disclosed	5.5	-	-	5.5 ± 0.9	>100	≥80	~0	-	-	[4]
3	Compound 2	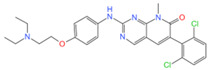	13.3	-	-	-	-	94	65	11	Significant	[48]
4	Compound 12	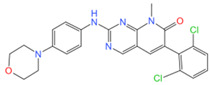	11.6	-	-	-	-	73	~65	36	None	[48]
5	Compound 16	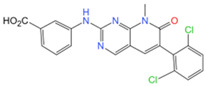	7.0	-	-	-	-	70	~57	47	None	[48]
6	Bosutinib isomer	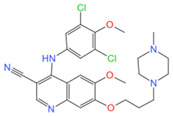	12.3	10	3	-	-	-	-	-	Significant *	[18]

Kinase inhibition rates were determined using compounds 3, 4, and 5 at 1 μM, and cleavage rates were measured at 10 μM. * The cellular proliferation IC_50_ values of Bosutinib isomer were 1.6 ± 0.19 μM in HEK293T cells. -: Data not provided in the cited literature.

### 8.1. Allosteric Inhibitors: GPHR-00336382 and GPHR-00355672

Using a full-length WEE2 homology model and the kinase domain crystal structure (PDB: 5VDK), researchers applied vHTS and HTS to comprehensive and allosteric-focused libraries [4]. After multiple rounds of validation to eliminate interfering and non-specific compounds, two highly selective allosteric WEE2 inhibitors were successfully identified: GPHR-00336382 and GPHR-00355672, with IC_50_ values against WEE2 of 5.8 ± 1.2 μM and 5.5 ± 0.9 μM, respectively. GPHR-00336382 binds to full-length WEE2 but not to the kinase domain, whereas GPHR-00355672 binds to the kinase domain. ELISA (Enzyme-Linked Immunosorbent Assay) results showed that both compounds reduced WEE2 activity by at least 80% but exerted almost no effect on WEE1 activity. Up to now, functional verification of the two inhibitors is limited to in vitro enzyme assays. Lack of cellular, embryonic and in vivo animal data leaves their cellular permeability, in vivo potency and toxicity uncharacterized.

### 8.2. ATP-Competitive Inhibitors

#### 8.2.1. Non-Selective Inhibitors

Hanna et al. identified potent and selective WEE2-targeted compounds using vHTS, molecular docking, and structural similarity analysis [48]. Compound 2 is a broad-spectrum protein tyrosine kinase inhibitor. ELISA assays confirmed that Compound 2 exhibited remarkable WEE2 inhibitory activity. Furthermore, biological activity evaluation demonstrated that Compound 2 significantly reduced embryonic cleavage rates, with only 11% of embryos initiating development. Notably, cell proliferation assays revealed that Compound 2 also displayed pronounced cross-inhibition against WEE1, thereby interfering with mitosis. Therefore, the inhibitor has low target specificity with severe off-target effects, cannot selectively regulate WEE2 function alone, and carries major safety hazards in vivo.

#### 8.2.2. Selective Inhibitors Targeting WEE2

Compounds 12 and 16 are both commercially available analogs of Compound 2 identified via similarity searching. ELISA assays confirmed that both compounds also reduce WEE2 activity. Biological activity tests showed that they decreased fertilization rates to 36% and 47%, respectively, while their effects on mitosis were negligible. These results indicate that both compounds represent promising candidates for further development or structural modification [48]. Like the aforementioned Compound 2, these two inhibitors have only been tested for activity in embryonic models without accurate IC_50_ quantification. The shortage of cellular kinetic and in vivo efficacy data warrants further scaffold optimization.

#### 8.2.3. Broad-Spectrum Kinase Inhibitors with Preferential Activity Against WEE2

A study confirmed that bosutinib isomer is a potent and selective broad-spectrum inhibitor with a preference for WEE2 [18]. Zhu et al. characterized its binding to WEE1, WEE2 and PKMYT1 using isothermal titration calorimetry (ITC) and found that it exhibits potent nanomolar affinity for WEE2 (K_d_ = 4.7 ± 2.3 nM), the strongest among the tested compounds. Thermodynamic and affinity analyses revealed a markedly enhanced specific binding preference for WEE2: its affinity for WEE2 is approximately 9.3-fold higher than for WEE1 (WEE1: K_d_ = 43.7 ± 10 nM), whereas binding to PKMYT1 is significantly reduced (K_d_ = 444 ± 41 nM), nearly 100-fold weaker than to WEE2, allowing efficient discrimination among the three kinases. Notably, it shows relatively weak antiproliferative activity at the cellular level, with an IC_50_ of 1.6–3.9 μM, and its in vivo utility may be limited by cellular permeability or its kinase selectivity profile. Therefore, these limitations may substantially restrict their subsequent in vivo animal studies and translational applications.

Three distinct strategies have emerged for WEE2 inhibitor development: (i) combined screening of allosteric-focused and comprehensive libraries; (ii) classical vHTS integrated with core scaffold derivatization and optimization; and (iii) repurposing known kinase inhibitors. Additionally, Shahid et al. recently identified candidate drugs including midostaurin and nilotinib through drug repurposing studies via virtual docking and molecular dynamics simulations [50]. Allosteric inhibitors target a unique WEE2 site with no cross-inhibition, while ATP-competitive inhibitors achieve selectivity via structural differences between WEE2 and WEE1 and side-chain modifications. Both categories show definite WEE2 inhibitory activity, laying a foundation for druggability optimization and clinical translation, and promise to provide novel non-hormonal options for female-controlled contraception.

## 9. Discussion

### 9.1. Functional Differentiation and Evolution of the WEE Family

WEE2, together with WEE1 and PKMYT1, belongs to the WEE kinase family. As an oocyte-specific WEE kinase, WEE2 exhibits clear functional divergence from the ubiquitously expressed WEE1 and PKMYT1. Both WEE2 and WEE1 are primarily localized in the nucleus, whereas PKMYT1 is mainly distributed in the endoplasmic reticulum and Golgi apparatus. In addition to blocking the G2/M transition [51,52], WEE1 can also participate in replication fork stability and S-phase checkpoint regulation by phosphorylating CDK2 [53,54]. PKMYT1 exhibits dual substrate specificity and functional redundancy, being capable of phosphorylating both Tyr15 and Thr14 residues simultaneously [55,56], and can specifically compensate for the loss of WEE1 function when WEE1 is impaired [57]. In contrast, WEE2 is not involved in somatic DNA damage repair or cell cycle regulation. Its core functions are confined to maintaining the timing of oocyte meiosis, promoting oocyte maturation, and initiating fertilization, with no functional redundancy with PKMYT1 [58].

In normal cells, WEE1 functions as a tumor suppressor. In tumor cells, however, WEE1 can exhibit “pseudo-oncogene” properties, and its high expression conversely becomes critical for cancer cell survival [59]. For example, WEE1 is highly expressed and exerts pro tumorigenic effects in multiple cancers including gastric cancer [60], leukemia, and melanoma [61]. Similarly, PKMYT1 is highly expressed and exerts pro-tumorigenic effects in most cancers, including chronic myeloid leukemia (CML) [62], breast cancer [63,64], and colorectal cancer (CRC) [65], but is downregulated and functions as a tumor suppressor in lung adenocarcinoma (LUAD) [66]. In addition, PKMYT1 also exhibits non-canonical functions in pancreatic cancer [67] and prostate cancer [68]. In contrast, WEE2 is not involved in tumorigenesis and has not been implicated in cancer development, invasion, or metastasis. Its absence results exclusively in female reproductive disorders, rather than somatic abnormalities or cancer susceptibility.

### 9.2. Effects of WEE2 Mutations on Protein Conformation and Functional Reversibility

Conformational changes induced by *WEE2* mutations generally lack reversibility, and the potential for functional restoration is highly dependent on mutation type, site, and extent of damage. Most pathogenic mutations that directly disrupt backbone folding or core functional structures—including frameshift, nonsense, and splicing mutations, as well as missense mutations affecting core catalytic residues or conserved hydrogen bonds—cause irreversible alterations, leading to complete loss of function and representing major genetic causes of human fertilization failure. Conventional AOA cannot reverse these deficiencies. In contrast, a small number of weakly pathogenic missense mutations that cause only mild local conformational disturbances (e.g., disruption of non-core hydrogen or ionic bonds without affecting overall kinase domain folding) can be rescued by exogenous interventions. For example, microinjection of wild-type *WEE2* cRNA into oocytes carrying such mutations enables expression of properly folded wild-type WEE2 protein, which functionally replaces the mutant variant and restores fertilization capacity. In vitro studies confirm that this approach supports morphologically normal blastocyst formation with no significant chromosomal abnormalities [16].

### 9.3. Challenges and Clinical Applications of WEE2-Targeted Inhibitor Development

While WEE2 loss in humans causes irreversible fertilization failure and full female sterility, two CRISPR-edited WEE2 knockout mouse strains with either a 17 bp deletion or a 21,878 bp large genomic deletion display markedly different reproductive traits. Both knockout mouse lines displayed normal growth, ovarian development, ovulation and estrous cycles. Only full-length knockout mice had slightly reduced litter sizes, without a complete infertility phenotype. Thus, mouse models fail to mimic human WEE2-mediated infertility and are unsuitable for assessing the in vivo contraceptive activity of WEE2 inhibitors [3]. Currently, the development of WEE2 inhibitors still faces critical technical bottlenecks. The limited screening scope of compound libraries makes it difficult to obtain candidates with high activity and affinity. The full-length structure of WEE2 remains unsolved, and its kinase domain lacks catalytic activity [18], rendering homology modeling and virtual screening unable to accurately reflect its true allosteric sites and conformational dynamics. Meanwhile, conventional cell systems hardly mimic the physiological microenvironment of oocytes; related experiments are costly and technically challenging. Therefore, it is urgent to establish a somatic cell-based high-throughput screening model to improve the efficiency and accuracy of validation. In clinical translation, there are also multiple challenges including off-target effects, toxic side effects, and the lack of a comprehensive evaluation system.

Early studies showed that there are species-specific conservation differences in the phosphorylation sites of WEE2, and its nuclear localization is critical for its function, making target positioning highly significant for drug design [69,70]. Studies have confirmed that the activity of porcine WEE2 entirely depends on the cAMP/PKA pathway [71], which is highly conserved among humans, pigs, and mice [6], suggesting that WEE2 can serve as a key pharmacological target. Currently, most WEE2 inhibitors target the ATP-binding pocket of the kinase catalytic domain. However, this pocket is highly conserved across the WEE kinase family, resulting in poor inhibitor selectivity and frequent off-target effects [23]. Functional homology in other domains further increases the risk of non-specific binding [18]. In addition, the selectivity data of existing inhibitors are only based on three kinases: WEE1, WEE2, and PKMYT3, lacking full verification of the entire kinase family. Therefore, non-specific binding to other kinases cannot be ruled out, indicating potential safety risks.

At present, inhibitors targeting WEE1 and PKMYT1 have entered clinical trials. WEE1 inhibitors (e.g., AZD1775) and PKMYT1 inhibitors (e.g., RP-6306) both induce prematurely mitotic entry and subsequent death of DNA-damaged cells [72,73]. Nevertheless, since both WEE1 and PKMYT1 are also expressed in normal cells, therapeutic administration is accompanied by adverse effects. Specifically, AZD1775 can severely impair bone marrow function and even induce aplastic anemia [74], whereas PKMYT1 inhibitors exhibit relatively mild toxicity, predominantly manifesting as rash and gastrointestinal reactions [75]. WEE1, PKMYT1 and WEE2 belong to the same gene family and share high structural and functional homology; accordingly, inhibitors with poor selectivity are prone to off-target cross-inhibition. For this reason, pregnant or fertile-age cancer patients receiving WEE1/PKMYT1 inhibitors must strictly follow medical advice to avoid reproductive toxicity. Similarly, insufficiently selective WEE2 inhibitors may also cause adverse reactions via off-target cross-inhibition. Therefore, great attention must be paid to the selectivity of WEE2 inhibitors in clinical applications, and standardized medication regimens should be strictly implemented to minimize risks.

## 10. Conclusions and Perspectives

As an oocyte-specific WEE kinase, WEE2 regulates meiosis and fertilization by phosphorylating CDK1 at Tyr15, and its structure determines its germline-specific function. Pathogenic mutations in *WEE2* are a major genetic cause of female infertility. Although some progress has been made in targeted inhibitors, issues such as limited molecules, insufficient activity, and low selectivity remain. Future efforts are needed to characterize its structure, improve the mutation spectrum, optimize inhibitors, and explore non-reproductive functions and gene therapies to promote precision diagnosis and treatment in reproductive medicine and improve female reproductive health.

## Data Availability

No new data were created or analyzed in this study.

## Abbreviations

The following abbreviations are used in this manuscript:

GV	Germinal vesicle
CDK1	Cyclin-dependent kinase 1
MPF	Maturation-promoting factor
MII	Metaphase II
WEE2	Wee1-like protein kinase 2
WEE1	Wee1-like protein kinase
PKMYT1	Membrane-associated tyrosine- and threonine-specific cdc2-inhibitory kinase
GVBD	Germinal vesicle breakdown
OMD	Oocyte maturation defects
TFF	Total fertilization failure
EDA	Early embryonic arrest
IVF	In vitro fertilization
IDRs	Intrinsically disordered regions
PKA	Protein Kinase A
cAMP	Cyclic Adenosine Monophosphate
cGMP	Cyclic Guanosine Monophosphate
CaM	Calmodulin
PDE3A	Phosphodiesterase 3A
CaMKII	Calcium/Calmodulin-Dependent Protein Kinase II
IP3	Inositol 1,4,5-trisphosphate
PIP2	Phosphatidylinositol 4,5-bisphosphate
EGF	Epidermal Growth Factor
LH	Luteinizing Hormone
CDC25A	Cell Division Cycle 25 Homolog A
CDC25B	Cell Division Cycle 25 Homolog B
CDC14B	Cell Division Cycle 14 Homolog B
APC-CDH1	Anaphase-Promoting Complex-Cdc20 Homolog 1
APC/C-CDC20	Anaphase-Promoting Complex/Cyclosome-CDC20
EMI1	Early Mitotic Inhibitor 1
14-3-3	Tyrosine 3-Monooxygenase/Tryptophan 5-Monooxygenase Activation Protein
ER	Endoplasmic Reticulum
Golgi	Golgi Apparatus
ICSI	Intracytoplasmic sperm injection
AOA	Artificial oocyte activation
GTEx	Genotype-Tissue Expression
AC	Aerobic capacity
MYC	MYC proto-oncogene
CHEK1	Checkpoint kinase 1
CHEK2	Checkpoint kinase 2
CDC25C	Cell division cycle 25 homolog C
CCNB1	Cyclin B1
OAD	Oocyte activation defect
PN	Pronuclear stage
NES	Nuclear export sequence
HTS	High-throughput screening
vHTS	Virtual HTS
ITC	Isothermal titration calorimetry
CML	Chronic myeloid leukemia
CRC	Colorectal cancer
LUAD	Lung adenocarcinoma
PIN1	Peptidylprolyl Cis/Trans Isomerase, NIMA-Interacting 1
